# Supplementary material for: Quantitative lung lesion features and temporal changes on chest CT in patients with common and severe SARS-CoV-2 pneumonia
Source: PLoS One. 2020 Jul 24;15(7):e0236858. doi: 10.1371/journal.pone.0236858 (PMC7380626; doi:10.1371/journal.pone.0236858)
Supplement: S1 Fig — (DOCX) [file pone.0236858.s001.docx]

**S1 Fig. Schematic diagram of AI calculation method for PGV, PCV and PTV.**


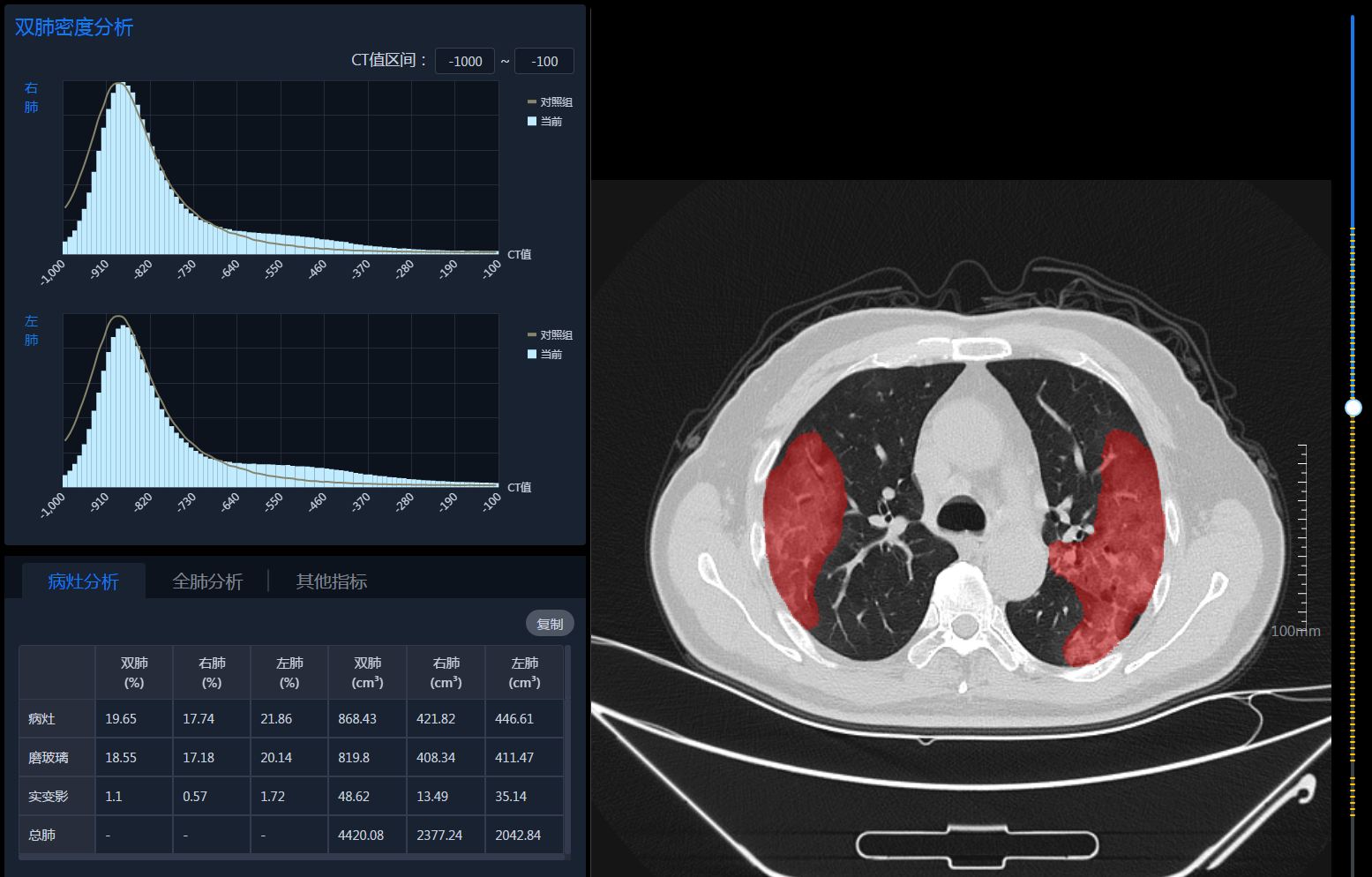


The distribution of CT values in lungs was calculated to obtain a histogram, and the quantitative parameters were subsequently used to compute PGV, PCV and PTV. AI, artificial intelligence; PGV, percentage of ground-glass opacity volume; PCV, percentage of consolidation volume; PTV, percentage of total pneumonia lesion volume.
