# Supplementary material for: Quantitative lung lesion features and temporal changes on chest CT in patients with common and severe SARS-CoV-2 pneumonia
Source: PLoS One. 2020 Jul 24;15(7):e0236858. doi: 10.1371/journal.pone.0236858 (PMC7380626; doi:10.1371/journal.pone.0236858)
Supplement: Attachment — Submitted filename: Response to reviewers.docx [file pone.0236858.s002.docx]

Specific points
1. For paired comparisons, there are quite a few of them and so there needs to be some correction of the level of significance to account for this large number of comparisons

*Re: Thank you for your comments. It is a very valuable advice. The question is also the limit in the study for not every patient included underwent CT scan in each stage. We have made a statistical analysis of all the data, and we use the exact p value to replace the p value in Mann-both Whitney U test and Wilcoxon test.*

2. Some of the p-values are much smaller than expected. For example, for severe disease, the total lung opacities for stage 3 was 21% (interquartile 14, 29) and for stage 4 was 20% (interquartile 8, 29.5), meaning that there was quite a big standard deviation and nearly identical median values. Further, the sample size n was only 20, yet the p-value comparison for stage 3 and 4 was a shockingly low 0.08, which is impossibly small for such data. Similarly the finding of consolidation was 5.5% with interquartile range 1, 8 for Stage 3 and was 3.5 with interquartile range 1.25, 5.75 for Stage 4 for severe disease, which with an n=20 should not yield a particularly significant p-value, but here is reported as p=0.003 which is surprisingly significant.

*Re: Thank you for your comments. These two sets of comparative data have been checked and analyzed repeatedly, and the p value in previous edition has been also replaced by the exact p value, but it has little change. We tried to analyze the comparisons by Mann-Whitney U test rather than paired comparisons, the p value was shockingly increased. Thus, we think that shockingly ow or particularly significant p value is because these two comparisons are paired tests.*

3. The manuscript needs to use precise terms that are defined clearly. Line 89, the “proportion of inflammatory volume” should probably be “percent lung volume involved by pathological opacity” or some other more specific term that includes the unit of measure. Oddly, the term “proportion of inflammatory volume” is never used again in the manuscript except fig 1. Instead the undefined ambiguous term “percentage of lung lesions” is used. What does “percentage of lung lesions”mean? Is it the percent of the diseased lung that is GGO? The total volume of the lung that is pathological in HU value? Please define.

*Re: Thank you for your comments. We are very sorry to confuse you by the inconsistent and ambiguous terms in our manuscript on lesion percentage. In previous version, both “proportion of inflammatory volume” and “percentage of lung lesions” mean “percentage of total lesion volume”. We have substituted these inconsistent terms by “the percentages of ground-glass opacity volume (PGV)”, “the percentages of consolidation volume (PCV)”, and the percentages of the total lesions volume (PTV)” in both lungs. The percentage of total lesions volume was defined with ranges of -700~-500 Hounsfield units (HU), that is, the sum area of both PGV and PCV.*

4. The HU ranges for normal versus GGO versus consolidation should be described – was it the same for all patients? Was it normalized or adjusted in any way for each patient? How exactly does the software assign the "proportion of inflammatory volume"?

*Re: Firstly, we have added it in the Material and Methods that percentages of lesion volume with ranges of -700~-500 Hounsfield units (HU), -500~60 HU, and* *-700~60 HU corresponded to percentages of ground glass opacity volume (PGV), consolidation volume (PCV) and total lesion volume (PTV), where total lesion volume was defined as the sum area of both PGV and PCV. The normal lung was defined with range of -1000~-700 HU. Secondly, data calculation method was the same for all patients. All patients with SARS-CoV-2 pneumonia were performed with the same CT scanner and the same scanning parameters. The data were also processed with the same software analysis parameters to ensure the consistency of these data. Thirdly,* *the software assigned the "proportion of inflammatory volume" by outlining the lesions in each layer of the scanned images, then acquired the volume of the lesions by calculating the pixels of each area outlined. The process also has been added in the Material and Methods.*

5. In the results, the term “percentage of lesions” is ambiguous and should be replaced by a more understandable term throughout the manuscript. Does it mean “lung volume percentage involved by pathological lung opacity” or is it just lung volume percentage involved by GGO or lung volume percentage involved by consolidation? The figures also use different terms. Fig 1 uses one term while Fig 2 uses the term “percentage of lung involvement” which is different than the terms used elsewhere in the text.

*Re: We are very sorry to confuse you by the inconsistent and ambiguous terms in our manuscript on lesion percentage. In previous version, percentage of lesions represented different means according to the conjunctions, eg. percentage of lesions of GGO or percentage of lesions of consolidation. Sometimes we omitted the conjunctions so that some confusion was produced. Thus, we have substituted these inconsistent terms by “the percentages of ground-glass opacity volume (PGV)”, “the percentages of consolidation volume (PCV)”, and the percentages of the total lesions volume (PTV)” in both lungs.*

6. The Y-axis of the figures needs to be the same between the three graphs (total, GGO, and consolidation). Having different y-axis makes it hard to compare.

*Re: Thank you for your comments. We have made the Y-axis of the figures the same between the three graphs (total, GGO, and consolidation). The modified figure has been uploaded.*
